# Prognostic Impact of the Neutrophil-to-Lymphocyte and Lymphocyte-to-Monocyte Ratio, in Patients with Rectal Cancer: A Retrospective Study of 1052 Patients

**DOI:** 10.3390/jpm10040173

**Published:** 2020-10-16

**Authors:** Zsolt Zoltán Fülöp, Réka Linda Fülöp, Simona Gurzu, Tivadar Bara, József Tímár, Emőke Drágus, Ioan Jung

**Affiliations:** 1 Department of Surgery, George Emil Palade University of Medicine, Pharmacy, Sciences and Technology, 540139 Targu Mures, Romania; zsolt_fulop15@yahoo.com (Z.Z.F.); rekafulop@ymail.com (R.L.F.); btibi_ms@yahoo.com (T.B.J.); 2 Department of Pathology, George Emil Palade University of Medicine, Pharmacy, Sciences and Technology, 38 Gheorghe Marinescu Street, 540139 Targu Mures, Romania; jungjanos@studium.ro; 3 Research Center (CCAMF), George Emil Palade University of Medicine, Pharmacy, Sciences and Technology, 540139 Targu Mures, Romania; 4 Second Department of Pathology, National Institute of Oncology, Faculty of Medicine, Semmelweis University, H-1085 Budapest, Hungary; jtimar@gmail.com; 5 Department of Urology, Clinical County Hospital, 540167 Targu Mures, Romania; d.emoke_29@yahoo.com

**Keywords:** neutrophil-to-lymphocyte ratio, lymphocyte-to-monocyte ratio, prognosis, rectal cancer, mesorectum, sphincter preserving

## Abstract

Despite the description of several new prognostic markers, colorectal cancer still represents the third most frequent cause of cancer-related death. As immunotherapy is considered a therapeutic alternative in such patients, neutrophil-to-lymphocyte (NLR) and lymphocyte-to-monocyte ratio (LMR) are hypothesized to provide reliable prognostic information. A retrospective study was conducted on 1052 patients operated on during 2013–2019 in two clinical hospitals from Hungary and Romania. Inclusion criteria targeted patients over 18 years old, diagnosed with rectal cancer, with preoperatively defined NLR and LMR. The overall survival rate, along with clinical and histopathological data, was evaluated. Overall survival was significantly associated with increased NLR (*p* = 0.03) and decreased LMR (*p* = 0.04), with cut-off values of 3.11 and 3.39, respectively. The two parameters were inversely correlated (*p* < 0.0001). There was no statistically significant association between tumor stage and NLR or LMR (*p* = 0.30, *p* = 0.06, respectively). The total mesorectal excision was especially obtained in cases with low NLR (*p* = 0.0005) and high LMR (*p* = 0.0009) values. A significant association was also seen between preoperative chemoradiotherapy and high NLR (*p* = 0.0001) and low LMR (*p* = 0.0001). In patients with rectal cancer, the preoperative values of NLR and LMR can be used as independent prognostic parameters. An NLR value of ≥3.11 can be used to indicate the response to preoperative chemoradiotherapy, but a low chance of sphincter preservation or obtaining a complete TME. Higher values of NLR and lower values of LMR require a more attentive preoperative evaluation of the mesorectum.

## 1. Introduction

Colorectal cancer (CRC) is responsible for about 10% of all diagnosed malignant tumors and cancer-related deaths worldwide [1]. Regarding its diagnosis between genders, it is the third most common cancer in men and the second most frequent in women [1]. Approximately one-third of CRC cases are diagnosed within the rectum [2].

In 1863, Rudolf Virchow first described a possible association between malignant tumors and inflammation, highlighting the role of the density of white blood cells in carcinoma behavior [3,4,5,6]. Recently, multiple studies have investigated the role of the systemic inflammatory response (SIR) in carcinogenesis, progression, and prognosis of different cancer types [6,7,8], but the results are controversial. The SIR is defined by several parameters, including the neutrophil-to-lymphocyte ratio (NLR) and lymphocyte-to-monocyte ratio (LMR). It is thought that NLR can predict prognosis, due to its close relationship with the cancer stage [3,7,9,10]. Increased preoperative NLR is caused by neutrophilia and/or lymphopenia, the two conditions of a pro-tumor inflammatory process. This value is, however, questioned because an elevated number of neutrophils indicates an acute SIR, whereas, cancers cause chronic SIR [5,11].

Chronic SIR is thought to be estimated by the LMR value [5]. In rectal cancer, in contrast to previously reported findings with NLR, it was observed that the preoperative LMR values were lower in patients with large tumors diagnosed in late stages [12]. Preoperative serum parameters are important for establishing treatment strategies [5]. Large cohorts are needed to establish the reliability of these cheap and easily quantified markers.

The present study aimed to validate the possible prognostic or predictive impact of preoperative NLR and LMR in a large cohort of patients with rectal cancer. The included patients underwent surgery in two university surgical departments, one from Romania and one from Hungary.

## 2. Materials and Methods

### 2.1. Patient Selection

The approval of the three Ethical Committees (Ethical Committee of the Clinical County Emergency Hospital and the Ethical Committee of the George Emil Palade University of Medicine, Pharmacy, Sciences and Technology, Targu Mures, Romania; Institutional Research Ethics Committee from Budapest, Hungary) was obtained for this study.

We performed a retrospective observational study that included all consecutive patients with rectal cancer who underwent surgery, between January 2013 and August 2019, in two university hospitals: The National Institute of Oncology of Budapest, Hungary, and the Emergency Clinical County Hospital of Targu Mures, Romania.

Besides preoperative serum values of NLR and LMR ratio, the following clinicopathological parameters were examined: Patient’s gender and age, the type of surgical procedure (with or without sphincter preservation), presence or absence of preoperative chemoradiotherapy (CRT), and tumor location (low vs. mid/upper rectum), along with the pTNM stage and overall survival rate (OS) rate. Patient follow-up ranged from 1 to 76 months.

Most of the patients received capecitabine/long-course radiation therapy or 5-fluorouracil (5-FU)/long-course radiation therapy (50 Gy in 28 fractions). Blood analyses were done one day before the surgical intervention or early in the morning on the day of surgery. To allow patient recuperation, an interval of 6–8 weeks passed between CRT and surgery.

The surgical approaches were abdominal-perineal excision of the rectum (APER), anterior resection (Dixon), and Hartmann’s resection. These interventions were performed by two highly experienced surgical teams in the two clinical centers.

The pathologists evaluated the macroscopical quality of total mesorectal excision (TME), which was scored as complete, partially complete, and incomplete. They also performed the microscopic evaluation of the depth of infiltration (pT stage), the quality of resection margins, the lymph node status (pN stage), and lymph node ratio (LNR) and established the pTNM stage [4].

Our study included patients over 18 years old, diagnosed with rectal cancer, with or without CRT, with preoperatively defined NLR and LMR, who underwent laparoscopic or open surgery. Exclusion criteria included biopsies, cases where death occurred less than one month postoperatively, patients with associated sepsis, autoimmune or hematologic diseases, and cases with incomplete available information.

### 2.2. Statistical Analysis

Data analysis was performed using Graph Pad Prism 7 and SPSS software. Nominal variables were characterized using frequencies. Quantitative variables were tested for normality of distribution using the Kolmogorov-Smirnov test and were characterized by median and percentiles (25th–75th) or by the mean and standard deviation (SD), as appropriate.

We used the Chi-squared test, Student’s t-test, Mann Whitney test, and Spearman correlation test. We used the cut-off value of 3.11 for NLR and 3.39 for LMR, respectively. The cut-off values were defined according to TME quality (1 was considered complete, and 0 was considered partially complete or incomplete). A receiver-operating characteristic (ROC) curve analysis was used to test the predictive power and to determine cut-off values for NLR and LMR. We estimated the OS using the Kaplan-Meier curves; log-rank tests were applied for pair-wise comparison of survival. To distinguish non-significant cofactors from significant independent predictors of OS, multivariate Cox proportional hazards regression analyses and backward stepwise elimination were used. The Cox model was adjusted for age, gender, pTNM stage, lymph node ratio (LNR), and distal resection margin quality. All tests were two-tailed tests, and a *p*-value < 0.05 was considered statistically significant.

## 3. Results

### 3.1. Clinicopathological Parameters

We retrospectively evaluated a database of 1052 patients with rectal cancer diagnosed over six years. Included patients had a mean age of 64.29 ± 11.32 (range 21–94) years. There was a male predominance, with 61.9% males and 38.1% females (M:F ratio was 1.62:1).

In three-quarters of the patients (74.8%), the tumor involved the middle/upper rectum (which encompassed an area 5–15 cm). The ratio between middle/upper and lower (<5 cm) rectum involvement was 2.96:1. As a consequence, sphincter-preserving surgery was done in 70.5% of patients; with a ratio of 2.39:1 (preserving vs. non-preserving sphincter). The ratio between the node-negative and node-positive cases was 1.39:1. A ratio of 2.20:1 was observed between locally advanced stages (pT3-4) and cases with a low level of infiltration (pT1-2) (Table 1).

**Figure 1:**
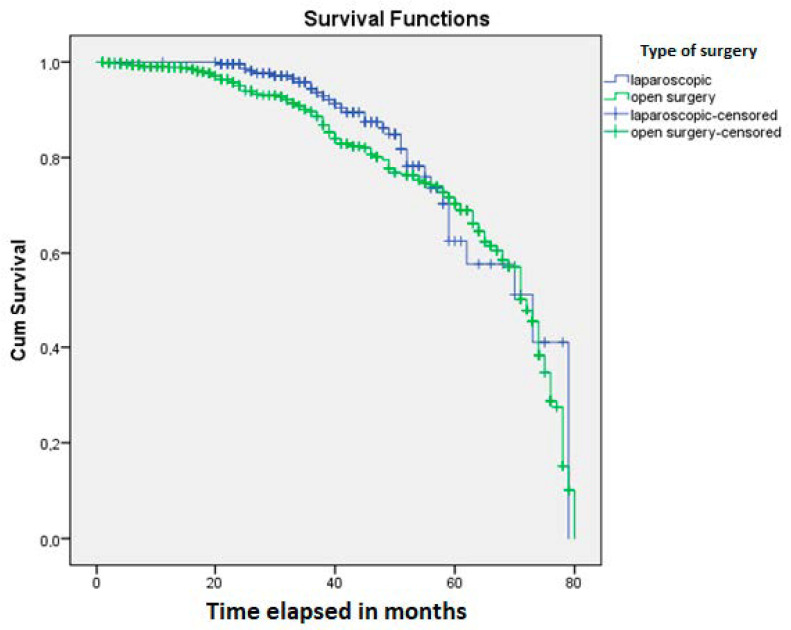
Patients with rectal cancer who underwent laparoscopic surgery did not show a longer overall survival than those who were treated by open surgery (*p* = 0.44).

The average time of the surgical interventions was 145 min. The laparoscopic surgeries required a significantly longer time (*p* < 0.0001), compared to the open interventions (162 vs. 128 min), without improving OS (*p* = 0.44) (Figure 1). However, significantly longer hospitalization was needed for patients who underwent open surgical intervention. Patients spent an average of two additional days in the hospital, compared with patients who underwent a laparoscopic intervention (*p* = 0.004). A significant difference was observed between open and laparoscopic approaches in the timing of the patient’s first stool in the postoperative period (*p* < 0.0001). The first stool was present one day earlier after laparoscopic interventions than after open surgery.

### 3.2. Particularities of the Systemic Inflammatory Response

An inverse correlation between LMR and NLR values was observed (*p* < 0.0001). This correlation was also highlighted when we compared the LMR and NLR values in males vs. females. Comparing with females, male patients showed slightly higher NLR and lower LMR values, independently by the patient’s age, especially for the cases of lower rectum who received preoperative CRT (Table 1).

As regarding NLR, independently from the depth of tumor infiltration, the NLR values were slightly lower in patients with tumors of the mid/upper rectum who did not show lymph node metastases and were treated with sphincter preserving, before receiving CRT (Table 1). Furthermore, there was no significant association between the clinical TNM tumor stage and NLR or LMR (*p* = 0.30, *p* = 0.06, respectively). LNR was not influenced by NLR (*p* = 0.13), but was associated with LMR value (*p* = 0.03).

A significant association was observed between the tumor distance from the anal verge and NLR (*p* < 0.0001) or LMR value (*p* < 0.0001), indicating that a longer distance from the anal margin results in a lower NLR and a higher LMR. The distance from the anal verge was directly associated with the tumor stage (*p* = 0.006). The lower rectum tumors presented mainly lower stages, while tumor perforation was more common when the tumor was localized at the upper level (*p* = 0.01). When CRT was initiated, the incidence of tumor perforation decreased considerably (*p* = 0.01). However, independent of tumor location (low vs. mid/upper rectum), a considerable number of cases (83.2%) from the low NLR group underwent sphincter-preserving surgery, in contrast with the high NLR group, where this rate was only 66.6% (Table 1).

LMR values were lower in patients with cancers of the lower rectum who responded to preoperative CRT and did not show lymph node metastases or deep infiltration (pT1-2N0 cases). In these patients, sphincter preservation was not frequently the therapy of choice (Table 1).

The integrity of the mesorectum following TME was significantly associated with the LMR (*p* = 0.0009), and the NLR value (*p* = 0.0005), same as with the total number of harvested lymph nodes (*p* = 0.01) and LNR (*p* = 0.04). In cases with higher NLR values, the integrity of the mesorectum was only partially complete or incomplete, reflecting an abundant tissue inflammation. In these cases, the value of the NLR did not significantly correlate with the duration of the surgery (*p* = 0.18, *r* = 0.06), although the surgery time was associated with the TME quality.

In those cases that required a shorter time of surgery, the TME was more frequently complete (*p* = 0.01), in contrast with difficult cases, which demanded a longer time. Obviously, TME quality was significantly associated with the tumor invasion of the circumferential resection margin invasion (*p* < 0.0001).

The above-mentioned correlations and associations showed that an NLR value of ≥3.11 can be used to indicate the response to preoperative CRT, but a low chance of sphincter preservation or obtaining a complete TME. Based on the same algorithm, an LMR value of ≥3.39 might indicate deep invasion or absence of preoperative CRT (Table 1).

### 3.3. Overall Survival

**Figure 2:**
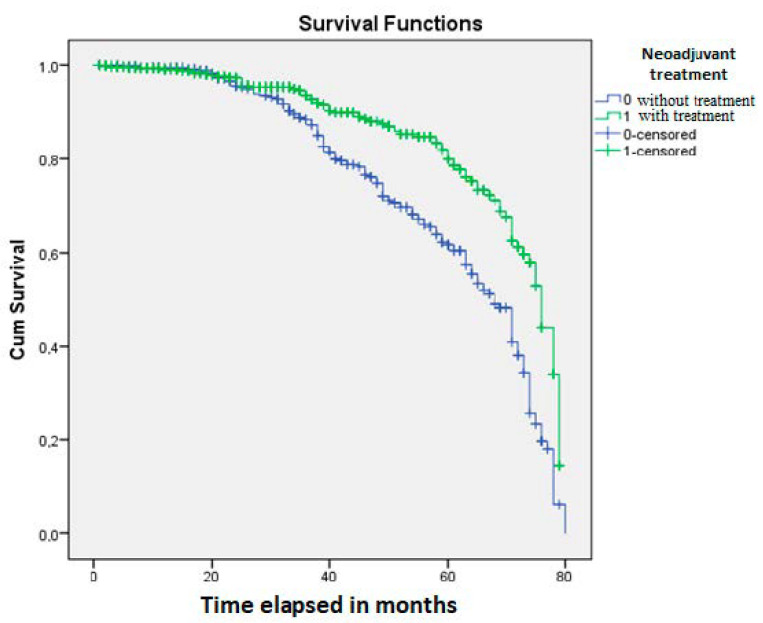
Neoadjuvant treatment significantly influences patients’ survival rate, compared to those who did not receive preoperative oncotherapy (*p* = 0.0001).

**Figure 3:**
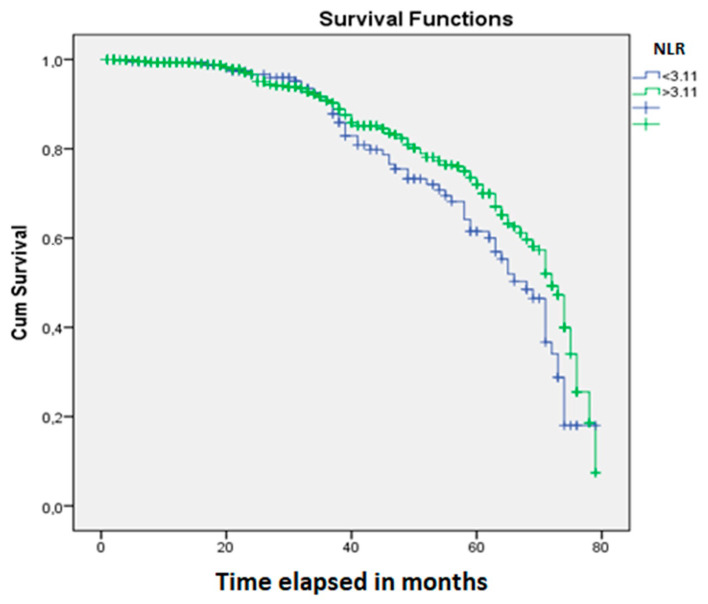
The cut-off value of 3.11 for the NLR can be used as an independent prognostic parameter for patients with rectal cancer (*p* = 0.03).

**Figure 4:**
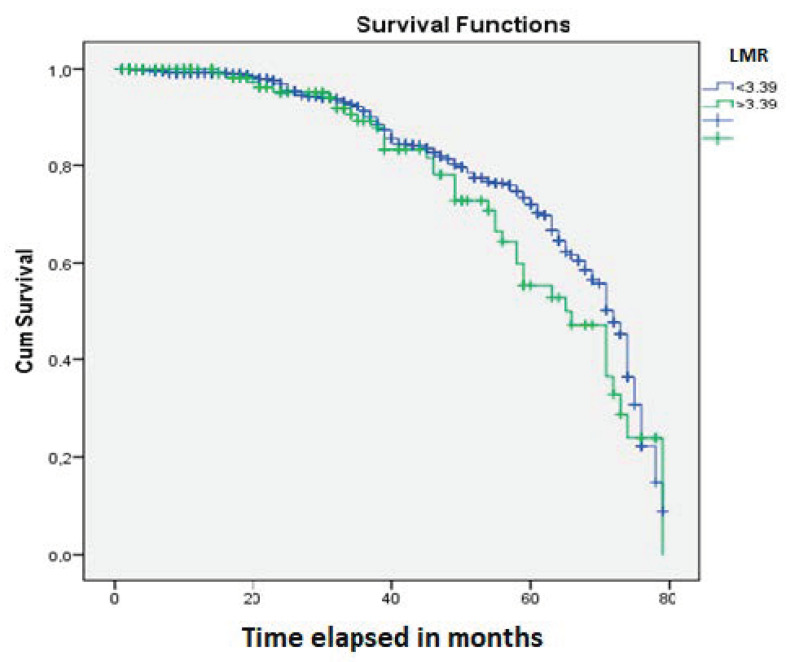
The cut-off value of 3.39 for the LMR can be used as an independent prognostic parameter for patients with rectal cancer (*p* = 0.04).

The Kaplan-Meier analysis showed a significant influence of neoadjuvant treatment on patients’ OS (*p* = 0.0001) (Figure 2). OS was found to be significantly influenced by SIR, defined by the cut-off values for NLR (*p* = 0.03) (Figure 3) and LMR (*p* = 0.04) (Figure 4).

Other parameters which significantly influenced patients’ survival rate were age group (*p* = 0.04) and gender (*p* = 0.007, male patients live longer), TNM stage (*p* < 0.0001), LNR (*p* < 0.0001), positivity of the distal resection margin (*p* < 0.0001), and tumor perforation (*p* < 0.0001). There was no statistically significant correlation between OS and tumor distance from the anal margin (*p* = 0.52).

## 4. Discussion

The immune response and SIR influence the rate of tumor growth and the risk of metastasis [3]. Strong tumor infiltration by inflammatory cells (including neutrophils) may contribute to intensified proliferation and tumor angiogenesis [4,9,12,13,14]. In these cases, the response to CRT may be altered [4].

As defined, the NLR is the ratio between the absolute number of neutrophils and the absolute number of lymphocytes [4,15]. Furthermore, LMR represents the absolute number of lymphocytes divided by the absolute number of monocytes [11]. Regarding NLR, an antitumor immunity suppressing role is attributed to neutrophils [15]. This suppression contributes to cancer progression, which is augmented by lymphocytopenia [3,14]. In contrast, high levels of tumor-infiltrating lymphocytes (TIL) are correlated with a longer OS [3,12]. This is attributed to cytotoxic activity and anti-angiogenetic cytokine production [4,11].

Although the investigated parameters (NLR, LMR) might be associated with the tumor stage, the literature data are controversial, and the underlying mechanisms of these results are not fully elucidated. For this reason, these ratios cannot yet consider independent prognostic factors [11,16]. They are influenced by several factors, which should be considered. Moreover, due to the behavior, anatomical topography, and therapeutic management of rectal cancer, the rate of inflammatory markers could have different relevance [13,17]. For example, an increased LMR might be induced by autoimmune or hematologic diseases, but also by infections [9], aspects that can explain the higher rate of incomplete TME in the cases with LMR ≥3.39.

The NLR and LMR values might also be influenced by preoperative CRT. Abe et al. demonstrated lower values for LMR in patients with tumors diagnosed in pT1-2N0 stages, in patients with rectal cancer who did not receive preoperative CRT, without correlation of LMR with tumor stage, after CRT [5]. Other authors, such as Caputo et al., have demonstrated no correlation with tumor stage [4], with a negative impact on OS [8,18] or, contrary, no impact of LMR on OS or disease-free survival [19]. Our apparently contradictory results, which proved at the limit of statistical significance (*p* = 0.05), lower LMR values for pT1-2N0 cases, are in line with data reported by authors, such as Mallapa et al. [16] or Abe et al. [5]. We did not find an association of LMR with LNR, which was defined as the number of positive lymph nodes divided by the total number of lymph nodes harvested [20,21], and not reported to the rectal lymph nodes only.

The controversial literature reports might be explained based on the number of examined cases (usually below 300), the used protocols therapeutically and the homogeneity of the cohort. As most of the authors perform statistical examinations in consecutive cases and establish “in-house” managed ratios, using receiver operating curve analysis [8], such in our material, controversial data might be influenced by these above-mentioned factors. It is necessary to mention, for example, that 68.8% of patients included in the present study were diagnosed in patients with pT3/4 staged tumors, with or without lymph node metastases, and correlations were done based on the cut-off value of 3.39. In contrast with other authors [7,16], we have included in the examined databases only rectal carcinomas, without tumors located in the colon or in the anal canal. On the other hand, some authors, such as Xiao et al. included only patients in the pT3N0 stage [18].

In patients with CRC, poorer OS was predicted when pre-treatment NLR showed elevated values [22]. In our material, the applied cut-off value for NLR was 3.11. An NLR of >3, before CRT/before surgery, is usually considered an indicator of poor prognosis, high recurrence rate, and low 5-year OS [4,15,23]. As regarding tumor stage, Caputo et al. reported, similar to our data, no significant association between the pTNM stage and NLR [4]. A higher clinical stage III rate was observed by Cha et al. in their cases within high NLR [11]. Other authors reported increased NLR in cases with positive nodal status on MRI [13,14]—this latter aspect being also proved by our data. Based on an in-house evaluation of large cohorts, a cut-off value should be established for prognostic evaluation [24,25]. In our material, NLR ≥3.11 and LMR <3.39 proved to be indicators of the response to preoperative CRT and lower risk for lymph node metastases.

Regarding TME, its quality was more frequent incomplete or only partially complete in cases with high NLR values. This aspect can be related to the surgical intervention quality or can reflect a peritumoral extensive inflammatory profile, which does not allow a proper and complete mesorectal excision. Scarce data can be found in the literature about the possible relationship between NLR and TME quality. Authors, such as Sung et al. [26] or Portal et al. [10], agree with the idea that pre-CRT and post-CRT NLR value might be used as a blood biomarker with a potential prognostic role in the case of patients who underwent curative TME intervention [10,26], but we did not find other supplementary explanation for this relationship. However, a high NLR and a low LMR value will lead in the majority of the cases to an incomplete TME quality, which will negatively influence patient’s OS, disease-free survival, and recurrence survival. In these circumstances, surgeons can identify preoperatively patients with a high risk of developing postoperative complications [4,27,28].

The strength of the present study consists of the investigation of one of the largest sample sizes so far, including only rectal cancer cases, from two medical centers. The limitations consist of its retrospective manner, and of including only two surgical centers, with predominantly locally advanced cases. These limitations were mitigated by using the largest databases reported in the literature, establishing an in-house designed value for both NLR and LMR. To elucidate the possible geographic-related differences, also reported for other cancers [29], a further multicenter study, in a larger cohort, should be performed.

## 5. Conclusions

The investigated parameters, NLR and LMR, are useful independent prognostic parameters. An NLR value of ≥3.11 can be used to indicate the response to preoperative CRT, but a low chance of sphincter preservation or obtaining a complete TME. An LMR value of ≥3.39 might indicate deep tumor invasion. The fact that NLR and LMR seem to influence the TME integrity is of great importance, which should be considered by rectal surgeons.

## Figures and Tables

**Table 1:** Correlation between clinicopathological factors and serum indicators of systemic inflammatory response.

Clinicopathological Parameters		Number n = 1052 (%)	NLR		*p* Value	LMR		*p* Value *
			<3.11 (23.9%)	≥3.11 (76.1%)		<3.39 (82.0%)	≥3.39 (18.0%)	
Gender	Male	(61.9)	(56.1)	(63.7)	0.05	(64.6)	(50.0)	0.001
	Female	(38.1)	(43.9)	(36.3)		(35.4)	(50.0)	
Age (years)	<60	(30.8)	(26.0)	(32.3)	0.09	(30.8)	(30.4)	0.92
	≥60	(69.2)	(74.0)	(67.7)		(69.2)	(69.6)	
Tumor location	Low	(25.2)	(14.8)	(27.0)	0.01	(28.2)	(7.7)	0.0001
	Mid/upper	(74.8)	(85.2)	(73.0)		(71.8)	(92.3)	
Depth of infiltration	pT1-2	(31.2)	(28.2)	(32.2)	0.30	(32.7)	(24.3)	0.05
	pT3-4	(68.8)	(71.8)	(67.8)		(67.3)	(75.7)	
Lymph node status	pN0	(58.2)	(60.0)	(50.0)	0.02	(60.0)	(50.0)	0.02
	pN+	(41.8)	(40.0)	(50.0)		(40.0)	(50.0)	
Sphincter-preserving	Yes	(70.5)	(83.2)	(66.6)	0.0001	(67.9)	(82.4)	0.0001
	No	(29.5)	(16.8)	(33.4)		(32.1)	(17.6)	
Preoperative oncologic therapy	Yes	(62.0)	(30.6)	(71.8)	0.0001	(68.0)	(34.5)	0.0001
	No	(38.0)	(69.4)	(28.2)		(32.0)	(65.5)	

(NLR = neutrophil-to-lymphocyte ratio; LMR = lymphocyte-to-monocyte ratio; * Chi square test).
